# Human airway tuft cells influence the mucociliary clearance through cholinergic signalling

**DOI:** 10.1186/s12931-023-02570-8

**Published:** 2023-11-04

**Authors:** Monika I. Hollenhorst, Thomas Husnik, Malin Zylka, Nele Duda, Veit Flockerzi, Thomas Tschernig, Stephan Maxeiner, Gabriela Krasteva-Christ

**Affiliations:** 1 Institute of Anatomy and Cell Biology, Saarland University, Homburg, Germany; 2 Institute for Experimental and Clinical Pharmacology and Toxicology, Preclinical Center for Molecular Signaling, Saarland University, Homburg, Germany

**Keywords:** Tuft cell, Brush cell, Airways, Human trachea, Trachea, Respiratory, Mucociliary clearance

## Abstract

**Background:**

Airway tuft cells, formerly called brush cells have long been described only morphologically in human airways. More recent RNAseq studies described a chemosensory cell population, which includes tuft cells, by a distinct gene transcription signature. Yet, until which level in the tracheobronchial tree in native human airway epithelium tuft cells occur and if they function as regulators of innate immunity, e.g., by regulating mucociliary clearance, remained largely elusive.

**Methods:**

We performed immunohistochemistry, RT-PCR and immunoblotting analyses for various tuft cell markers to confirm the presence of this cell type in human tracheal samples. Immunohistochemistry was conducted to study the distribution of tuft cells along the intrapulmonary airways in humans. We assessed the influence of bitter substances and the taste transduction pathway on mucociliary clearance in mouse and human tracheal samples by measuring particle transport speed.

**Results:**

Tuft cells identified by the expression of their well-established marker POU class 2 homeobox 3 (POU2F3) were present from the trachea to the bronchioles. We identified choline acetyltransferase in POU2F3 expressing cells as well as the transient receptor potential melastatin 5 (TRPM5) channel in a small population of tracheal epithelial cells with morphological appearance of tuft cells. Application of bitter substances, such as denatonium, led to an increase in mucociliary clearance in human tracheal preparations. This was dependent on activation of the TRPM5 channel and involved cholinergic and nitric oxide signalling, indicating a functional role for human tuft cells in the regulation of mucociliary clearance.

**Conclusions:**

We were able to detect tuft cells in the tracheobronchial tree down to the level of the bronchioles. Moreover, taste transduction and cholinergic signalling occur in the same cells and regulate mucociliary clearance. Thus, tuft cells are potentially involved in the regulation of innate immunity in human airways.

**Supplementary Information:**

The online version contains supplementary material available at 10.1186/s12931-023-02570-8.

## Background

The airway epithelium is composed of various cell types. Their identities and functions have more recently been defined using sequencing studies of mouse and human airway epithelium [1–4]. Among those are the rare tuft cells, formerly referred to as brush cells in the lower airways and as solitary chemosensory cells in the upper airways [5]. These cells have over decades merely been described morphologically by the presence of their apical tuft of microvilli [6]. They are classified as a rare population of cells, given that they constitute approx. 1% of the total epithelial cell population in mice [7, 8]. Despite their scarcity tuft cells in the airways are chemosensory and execute essential functions as triggers of fundamental innate immune processes as this has become evident more recently [5]. However, most functional studies on tuft cells have been performed in mouse models, and little is known about their distribution and function in human airways.

One hallmark of these cells is their expression of choline acetyltransferase (ChAT), an enzyme responsible for the synthesis of acetylcholine (ACh) [1, 9, 10]. Additionally, these cells express members of the bitter taste signalling cascade in mice, which is known to convey detection of bitter substances in taste buds. Activation of tuft cells with bitter substances, such as denatonium or bacterial quorum-sensing molecules with bitter character, leads to an increase in the intracellular Ca^2+^ level [11, 12]. The increased intracellular Ca^2+^-level then activates the transient receptor potential melastatin 5 (Trpm5) cation channel, which leads to downstream effects, such as the release of acetylcholine (ACh) from mouse tracheal epithelial tuft cells [11, 13]. Alternatively, Trpm5-dependent ACh release may be induced by other bacterial products, such as formyl peptides or succinate, independent of bitter taste receptors [14, 15]. In mice, the released ACh acts in a paracrine manner by inducing protective innate immune responses such as stimulation of mucociliary clearance [11, 14] as well as evoking protective neurogenic inflammation [12, 16], which proved to be essential to overcome bacterial infections with *Pseudomonas aeruginosa* [12].

However, in humans, tuft cells are less well characterised, since most studies, in particular functional ones, have been conducted in mice. Shah et al. reported an increase in ciliary beat frequency (CBF) after stimulation of bitter taste receptors with the bitter substance denatonium in primary airway epithelial cell cultures from human trachea and bronchi [17]. In sinonasal primary epithelial cell cultures *P. aeruginosa* quorum-sensing molecules were shown to stimulate CBF dependent on the bitter taste signalling component PLC_β2_ and on nitric oxide (NO) [18]. Yet, these effects observed on CBF after stimulation of bitter taste signalling in the lower and upper airways were attributed to bitter taste receptors present in ciliated cells and the role of tuft cells was not addressed in both studies. Moreover, presence of characteristic markers in human airway tuft cells have mostly been described on RNA levels by RNAseq studies and not on protein level [2–4]. Here, we investigated the presence of tuft cells along the lower respiratory tract in human body donor specimens. Additionally, we characterised the cells and assessed the occurrence of gene transcripts in humans from molecules involved in tuft cell functions in mice. Moreover, we delineated the impact of tuft cells on mucociliary transport in native human tracheal epithelial preparations.

## Methods

### Human and mouse sample preparation

All body donors had previously given their consent and all procedures were approved by the Ethics Committee of Saarland Medical Association. For histological evaluation, samples were prepared from lungs dissected from body donors within 24 h after death. Prior to their death, all body donors had signed an agreement to bequeath their bodies to the Institute of Anatomy and Cell Biology after passing away. Only body donors who died of causes of death unrelated to airway diseases such as pneumonia or COPD were included in our study. Tissue specimens from different regions (trachea, main bronchi, lobar bronchi, segmental bronchi, bronchioles as well as alveolar regions) were collected and further processed for fixation (see below).

Tracheal samples were collected for particle transport speed (PTS) measurements, nucleic acid and protein extraction as follows. The upper 3–4 cartilage rings of the trachea located distal the cricoid cartilage were dissected and immediately transferred into cold media (MEM: minimal essential medium (Gibco, Thermo Fisher Scientific, Waltham, MA, USA) supplemented with L-glutamine (Gibco) and penicillin/streptomycin (Gibco). The epithelial layer was carefully separated from the conductive tissue, cut into small pieces and incubated at 37 °C and 5% CO_2_ in MEM until PTS measurements. Human tissue samples for nucleic acid (RNA, DNA) and protein extraction were collected within 18 h after death in RNeasy sample buffer, DNA lysis buffer, and RIPA-buffer, respectively. For this purpose, the epithelial layer was separated from the remaining tracheal wall by a rubber cell scraper (Sarstedt, Nümbrecht, Germany).

Experiments on mice were performed using wild type or *ChAT*-eGFP [19] mice older than 12 weeks of both sexes. All animal procedures were conducted in accordance with the German guidelines for care and use of laboratory animals. Mouse tracheas were prepared as described previously [11] and used for RNA extraction or PTS measurements. For RNA extraction and immunoblotting analyses of the epithelium, the epithelium was scraped off with a rubber cell scraper, and collected in respective buffers (see above).

### RT-PCR analysis

Human and mouse tissue samples were harvested, and RNA was extracted using the RNeasy Mini Kit (Qiagen, Hilden, Germany) according to the manufacturer’s protocol. DNA was isolated by tissue lysis and subsequent nucleic acid precipitation using 2-propanol as has been described previously [20]. All extracted nucleic acid preparations (i.e., RNA and DNA) were quantified using a NanoDrop One (Thermo Scientific, Waltham, MA, USA). DNaseI treatment of isolated RNA as well as subsequent cDNA synthesis was performed as follows: 1 µg of isolated total RNA, or in case of lower concentrations a maximal volume of 8 µl, were incubated with 1 µl 10x DNaseI Reaction buffer and 1 µl DNaseI (amplification grade, both Thermo Scientific) in a total volume of 10 µl (add DNase- and RNase-free water if applicable) for 15 min at 37 °C. To assure thorough DNA digestion, reactions were further supplemented with another 1 µl of DNaseI and incubated for additional 45 min before enzymatic reaction was terminated by addition of 1 µl 25 mM EDTA solution and incubation at 65 °C for 10 min (all reagents were from Thermo Scientific). In the next step, DNaseI-treated RNA samples were subject to reverse transcription following the manufacturer’s protocol using the SuperScript II reverse transcription kit (Thermo Scientific). Briefly, 10 µl of the DNaseI-treated RNA samples were incubated with 1 µl Oilgo dT_18_ Primer and 1 µl dNTPs (10 mM, each; both Thermo Scientific) for 5 min at 65 °C, before adding 4 µl First Strand Buffer (5x) and 2 µl DTT (0.1 M). Samples were incubated for 2 min at 42 °C before the reverse transcription was initiated by adding 1 µl of SuperScript II Reverse Transcriptase. The enzyme was substituted by 1 µl RNase/DNase-free water in control reactions. Reactions were incubated for 50 min at 42 °C and eventually terminated by an incubation at 72 °C for 15 min. All reactions yielded a total volume of 20 µl and were subsequently used for PCR analysis.

Gene transcription was assessed using the SYBR®Green-based, two-primer detection method. Primer pairs for the detection of cDNA of gene transcripts from mice have been published before [11] and were purchased from (Eurofins Scientific, Luxembourg City, Luxembourg). Primer pairs for the detection of human gene transcripts have been purchased from IDT (Integrated DNA Technologies, Coralville, IO, USA) employing the company’s respective PCR design tools (https://eu.idtdna.com/pages/tools). A list of the primer sequences and the access numbers of the underlying nucleotide sequence from which they derived can be found in Suppl. Table 1. All PCR reactions were run using the Bio-Rad CFX Connect™ RealTime System with its software (Bio-Rad Laboratories, Inc., Hercules, CA, USA). Sample preparation for PCR was set up as follows: 10 µl iTaq Universal SYBR®Green Supermix (Bio-Rad), 8 µl of nuclease-free water, 1 µl of cDNA (or control) of the previous step (alternatively 1 µl of 5 ng/µl genomic DNA sample) and 1 µl of a gene-specific primer mix. Primer mixes were prepared by the combination of gene-specific forward and reverse oligonucleotides at a final concentration of 10 mM. Reactions of a total volume of 20 µl were loaded on 96-well plates suitable for the CFX Connect™ RealTime System. Reactions were run according to the manufacturer’s protocol, i.e., initial denaturation at 95 °C for 30 s followed by a repetition of 40 cycles at 95 °C for 5 s and 60 °C for 30 s. PCR products were supplemented with OrangeG-colored loading-dye, separated on a 2% agarose gel and documented using a ChemiDoc™ XRS + with Image LabTM Software (Bio-Rad).

### Immunoblot analysis

Tissue samples were collected in RIPA-buffer (10 mM sodium phosphate buffer, 40 mM sodium fluoride, 2 mM EDTA, 0.1% sodium dodecyl sulfate, 1% TritonX-100, 0,1% sodium deoxycholate) supplemented with a proteinase inhibitor cocktail (cOmplete, Roche Diagnostics GmbH, Mannheim, Germany) and homogenised using a speed mill. Samples were incubated for 2 h at 4 °C to allow protein solubilization and centrifuged at 4000x g for 10 min at 4 °C. The supernatant was removed and its protein concentration was determined using a BCA-Protein Assay Kit (Thermo Scientific). A total of 40 µg protein per sample was diluted in SDS-sample buffer, loaded and separated by SDS-gel electrophoresis on a 10% acrylamide gel. Subsequently, gels were blotted on a nitrocellulose membrane and blocked in 5% milk powder dissolved in TBS-T buffer (Tris-buffered saline: 8.5 mM Tris-HCl, 1.7 mM Tris, 50 mM NaCl, 0.1% Tween-20) for 1 h. Membranes were further incubated in blocking buffer supplemented with respective primary antibodies at 4 °C overnight. On the following day, membranes were washed three times for 10 min in TBS-T buffer before they were incubated in blocking buffer supplemented with the secondary antibody for 1 h at room temperature. After another round of three washing steps in TBS-T buffer for 10 min at room temperature, membranes were subject to enhanced chemiluminescence reaction using the Supersignal West Pico Plus ECL Kit (Thermo Scientific) according to manufacturer’s recommendation. ECL-reaction was documented using a Bio-Rad geldoc system. The following antibodies have been used: CHAT (host rabbit monoclonal 13H9L16; concentration 1:1000; Merck), POU2F3 (host rabbit polyclonal AV32537; concentration 1:1000; Sigma-Aldrich, St. Louis, MO, USA) and anti-rabbit horseradish peroxidase-coupled secondary antibody (anti-rabbit IgG; concentration, 1:20,000; Sigma-Aldrich, Steinheim, Germany).

### Histology and immunofluorescence analysis

**Fig. 1:**
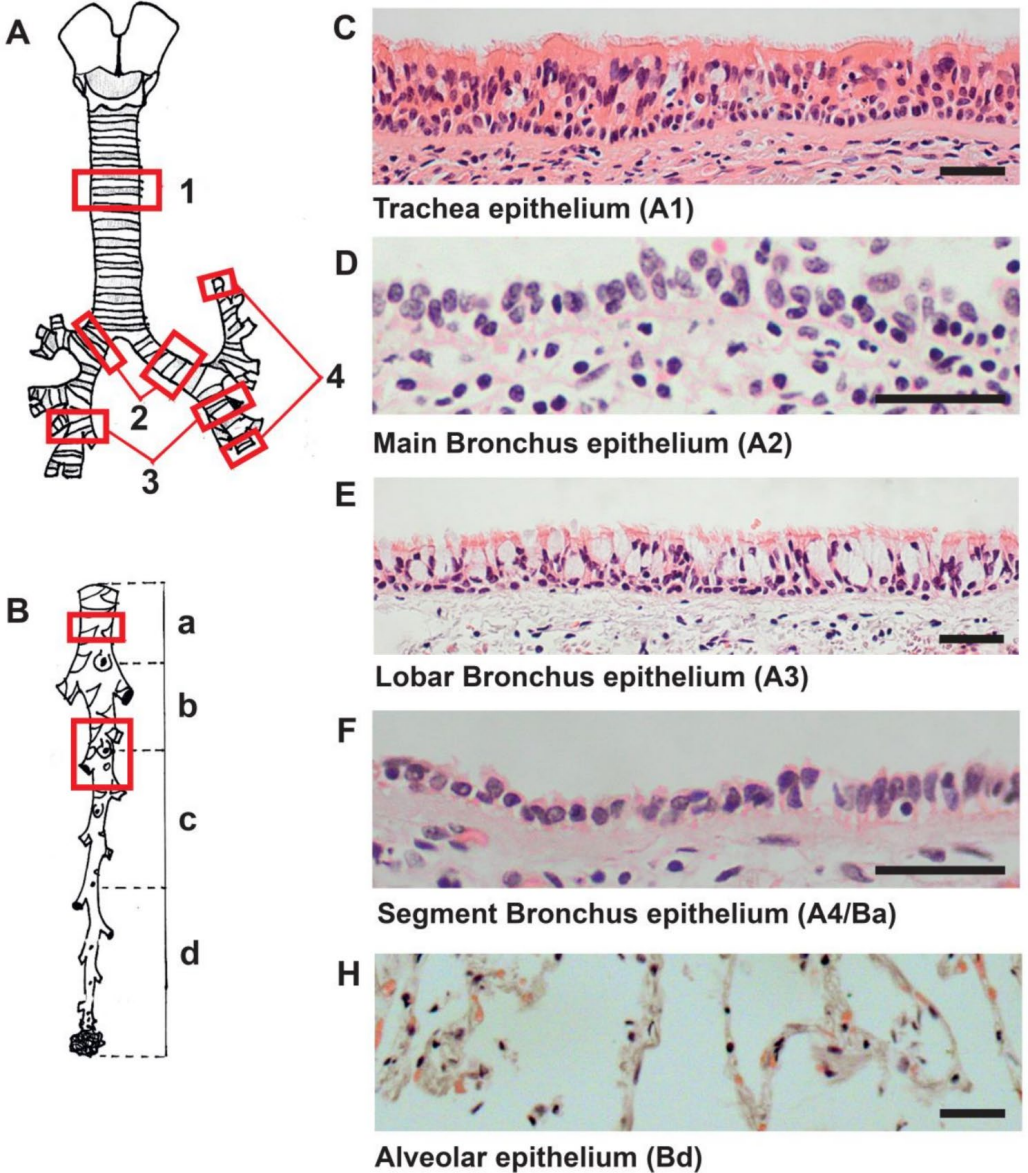
Overview of the human lower airways. (**a, b**) Schematic representation depicting different parts of the lower airways, red frames: trachea (A1), main bronchus (A2), lobar bronchus (A3), and segmental bronchus (A4/Ba) and bronchioles with alveolar region (Bc). (**c-h**) Representative eosin and hematoxylin stained tissue sections of tracheal epithelium, main bronchial epithelium, lobar bronchial epithelium, segmental bronchial epithelium and alveolar region. Scale bar: 100 μm

Fixation of the tissue and processing for immunofluorescence analysis have been performed as described previously [12]. Briefly, dissected tissues were subject to fixation in Zamboni fixative, cryo-preserved in Tissue Tek und stored at -20° C until use. Samples for bright field documentation upon classical hematoxylin/eosin staining (HE) were subject to dehydration and paraffine-embedding. Paraffine-embedded samples were sectioned at 10 μm, collected on glass slides, hydrated, processed for HE-staining, dehydrated, mounted in Mowiol (Sigma-Aldrich), and documented by bright field microscopy (Fig. 1b/c). Prior to blocking of unspecific antigens antigen-retrieval was performed with cryosections in a boiling 10 mM sodium citrate buffer (pH 6) for 5 min. The following primary antibodies have been used to detect CHAT (host, goat; concentration, 1:800; Merck Millipore, Temecula, CA, USA), DCLK1 (host, rabbit; concentration, 1:1600; abcam, Cambridge, UK), POU2F3 (host, rabbit; 1:1600; Sigma-Aldrich, St. Louis, MO, USA), PGP9.5 (host, rabbit; concentration 1:800; abcam) and TRPM5 (host rabbit, 1:200; Proteintech, Manchester, UK for human tissues and Trpm5 794 [11] host, rabbit, 1:800, for mouse tissues) followed by the application of secondary antibodies, Cy3-donkey anti-rabbit (concentration, 1:500; Merck Millipore), Cy3-donkey anti-goat (concentration, 1:400; Merck Millipore), Cy5-donkey anti-rabbit (concentration, 1:250; Jackson ImmunoResearch, Cambridge, UK). All sections were mounted using Mowiol (Sigma-Aldrich) and evaluated using a Zeiss epifluorescence microscope (AxioImager M2 with Axio-Cam 512 color, Zeiss, Oberkochen, Germany) equipped with ZEN software (Zeiss) for documentation.

### Measurements of particle transport speed in human tracheal preparations

For PTS experiments, tracheal epithelial preparations were pinned into a Sylgard-coated (Dow Corning GmbH, Wiesbaden, Germany) delta t-dish (Bioptechs, Butler, PA, USA) with the luminal side upwards. The delta t-dish dish contained 1.5 ml pre-heated buffer solution consisting of (in mM): 136 NaCl, 5.6 KCl, 10 Glucose, 10 HEPES, 1 MgCl_2_, 2.2 CaCl_2_, pH 7.4. To perform the measurement, the delta t-dish was mounted under an Eclipse 80i microscope (Nikon, Tokio, Japan) equipped with a SMX16E1M camera (Sumix, Oceanside, CA, USA) and 1–3 µl dynabeads (Invitrogen, Thermo Fisher) were added to the dish. Videos were recorded using the Streampix 7 software (Norpix Inc, Montreal, Canada) at every two minutes, starting from minute 0 until minute 29. Baseline was recorded until minute 7 and at minute 8 the first substance was applied. ATP (Sigma-Aldrich) was added in each experiment at minute 24 to assess the viability of the tissue. The following substances were used to investigate their influence on the transport speed of the dynabeads (particles): denatonium (1 mM, Sigma-Aldrich), quinine (100 µM, Sigma-Aldrich) and ATP (100 µM). The following inhibitors were applied: TPPO (100 µM, Sigma-Aldrich), atropine (50 µM, Sigma-Aldrich), mecamylamine (100 µM, Sigma-Aldrich) and L-Name (20 µM, Enzo Life Sciences, Lörrach, Germany). PTS was evaluated by tracking each particle in the videos using the ImageProPremier 9.3 software (Media Cybernetics Inc, Rockville, MD, USA).

### Measurements of particle transport speed in mouse trachea

Mouse tracheal PTS measurements were performed as described previously [11]. Mice were sacrificed by inhalation of an overdose of isoflurane followed by aortic exsanguination and tracheae were immediately dissected and opened longitudinally. The measurement was performed as described above for human PTS measurements. Videos were recorded starting 30 min after exsanguination. The following substances were used: denatonium (1 mM), diphenidol (200 µM, Sigma-Aldrich), chlorpheniramine (300 µM, Sigma-Aldrich), and TPPO (100 µM).

### Statistical analyses

PTS measurements were repeated at least with n = 4 samples from a minimum of 3 body donors. Immunohistochemistry was performed on tracheal and bronchial preparations from at least 3 different body donors. To assess statistical differences in the frequency of airway tuft cells, data were subjected to one-way ANOVA followed by Tukey’s post-hoc analyses for multiple comparisons. To assess statistical differences in PTS, the paired Student’s *t*-test was applied after the data were subjected to a Kolmogorov-Smirnov analysis to test for normal distribution. P values < 0.05 were considered statistically significant. All statistical analyses were performed using the GraphPad Prism 9 software (GraphPad Software, Boston, MA, USA).

## Results

### Presence of tuft cells in various segments of the lower airways

To determine the presence and location of tuft cells in the human epithelium of the lower respiratory tract, tissue samples of human body donors have been harvested and processed for immunofluorescence analyses. Various regions were chosen to prove for a potential far-ranging distribution of chemosensory cells in the human respiratory tract. Samples were collected from five different parts of the respiratory tract (Fig. 1a): (1) the trachea, (2) the main bronchus, (3) the lobar bronchus, (4) the segmental bronchus and (5) the bronchiolus with the alveolar region.

**Fig. 2:**
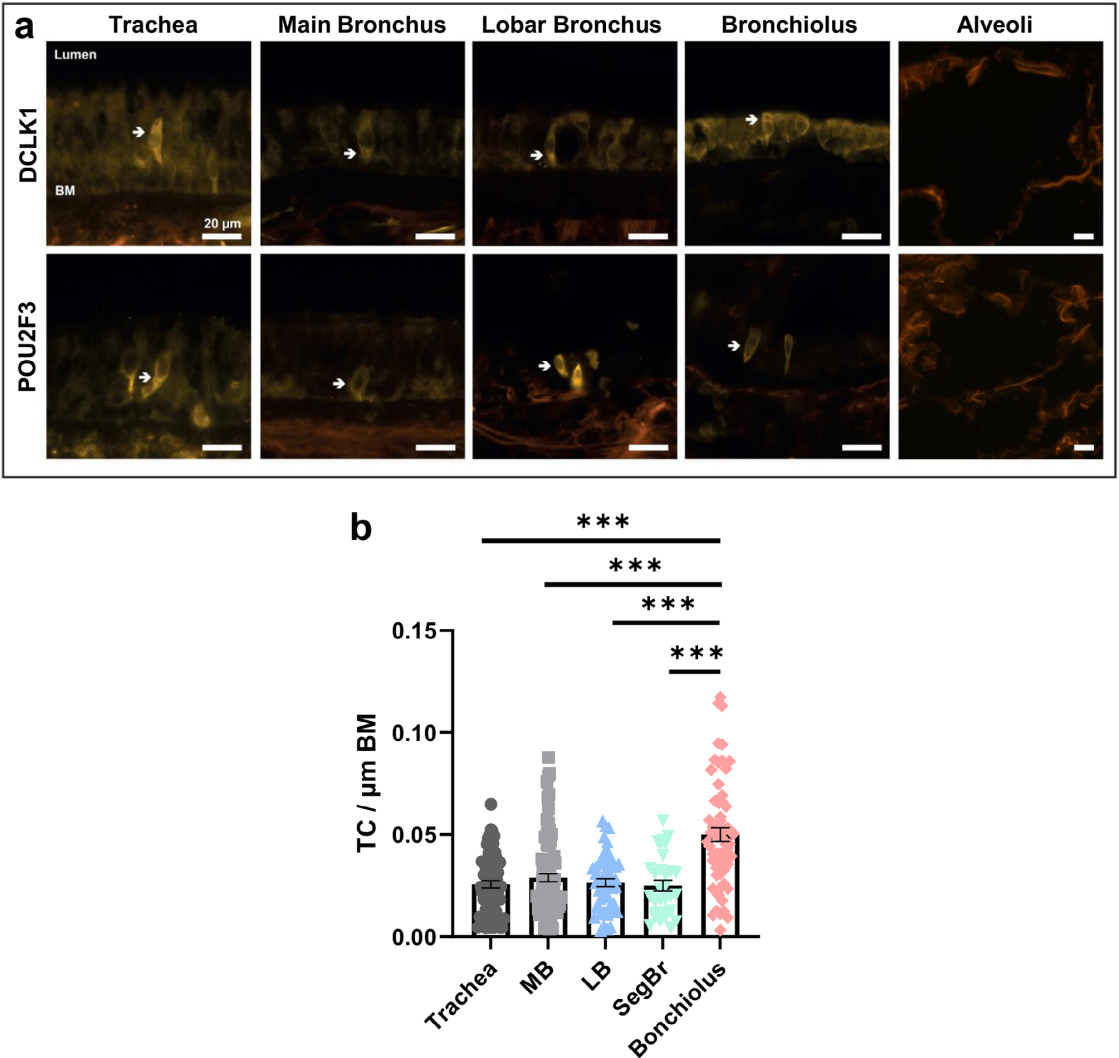
Immunofluorescence staining of selected tuft cell marker proteins in the human lower airways. (**a**) Immunolabeling for the tuft cell markers, DCLK1 (doublecortin like kinase 1) and POU2F3 (POU class 2 homeobox 3) indicates that tuft cells are present (white arrow) in all depicted parts of the lower airways, such as the trachea, main bronchus, lobar bronchus and bronchiolus but not in the alveolar region. (**b**) Quantification of tuft cells revealed a higher frequency of these cells in bronchioles compared to the trachea and bronchi (*** p < 0.001, n = 31–92 representative pictures from four body donors). BM, basal membrane. MB: Main Bronchus, LB: Lobar Bronchus, SegBr: Segment Bronchus, Scale bar: 20 μm

We applied a human and a mouse tuft cell marker to assess the distribution of tuft cells in the lower airways. POU class 2 homeobox 3 (POU2F3) was detected in human tuft cells in sequencing studies of human airway epithelium [4]. The doublecortin like kinase 1 (DCLK1) is an established marker for mouse tracheal tuft cells [1]. We identified POU2F3^+^ or DCLK1^+^ cells with the morphology of tuft cells in the human tracheal epithelium of the main, lobar and bronchioles, whereas no POU2F3^+^ or DCLK1^+^ cells were detected in the peripheral lung tissue of the alveolar region (Fig. 2a). Quantification of the tuft cells in the different regions of the respiratory tree from 4 body donors revealed a significantly increased frequency of these cells in the bronchioles compared to the trachea and bronchi (Fig. 2b). Results were similar across all body donors.

### Gene transcription of tuft cell markers in mouse and human tracheal epithelium

**Fig. 3:**
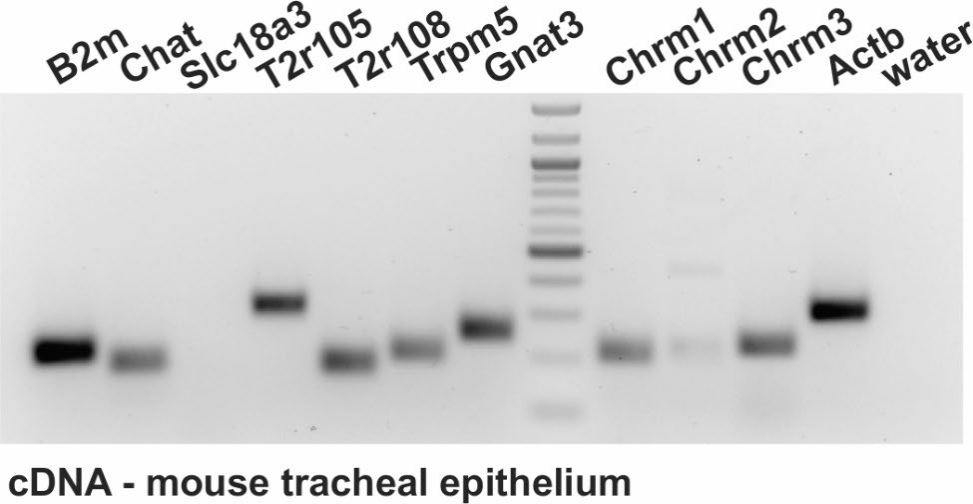
Gene transcription profile of mouse tracheal epithelium. The gene transcription profile in mouse tracheal epithelial samples. Beta-microglobulin (B2m) and Actb (actin beta) served as positive controls. Components of the bitter taste signaling cascade were present in mouse tracheal epithelium, such as Chat, T2r105/108, Gnat3 (alpha-gustducin), as well as muscarinic ACh receptors 1 and 3 (Chrm1/3). A faint signal was observed for Chrm2 and a band representing solute carrier family 18 member a3 transcripts (Slc18a3 also known as ‘Vacht’, vesicular acetylcholine transporter) was not detected. Input controls of RNA without reverse transcription and genomic controls for each assay can be found in Suppl Fig. 1

Given the number of established chemosensory markers being expressed in mouse tracheal epithelium, we investigated the transcription of genes involved in bitter and cholinergic signalling employing RT-PCR from mouse epithelial RNA samples (Fig. 3), before expanding our investigation to human tracheal epithelium. We were able to identify Chat, Tas2r105 and Tas2r108, Gnat3, and Trpm5, as well as muscarinic ACh receptors (mAChR) 1 and 3 (Chrm1/Chrm3). The results were validated by controls including genomic DNA as well as RNA samples that have not been subject to reverse transcription (Suppl. Figure 1).

**Fig. 4:**
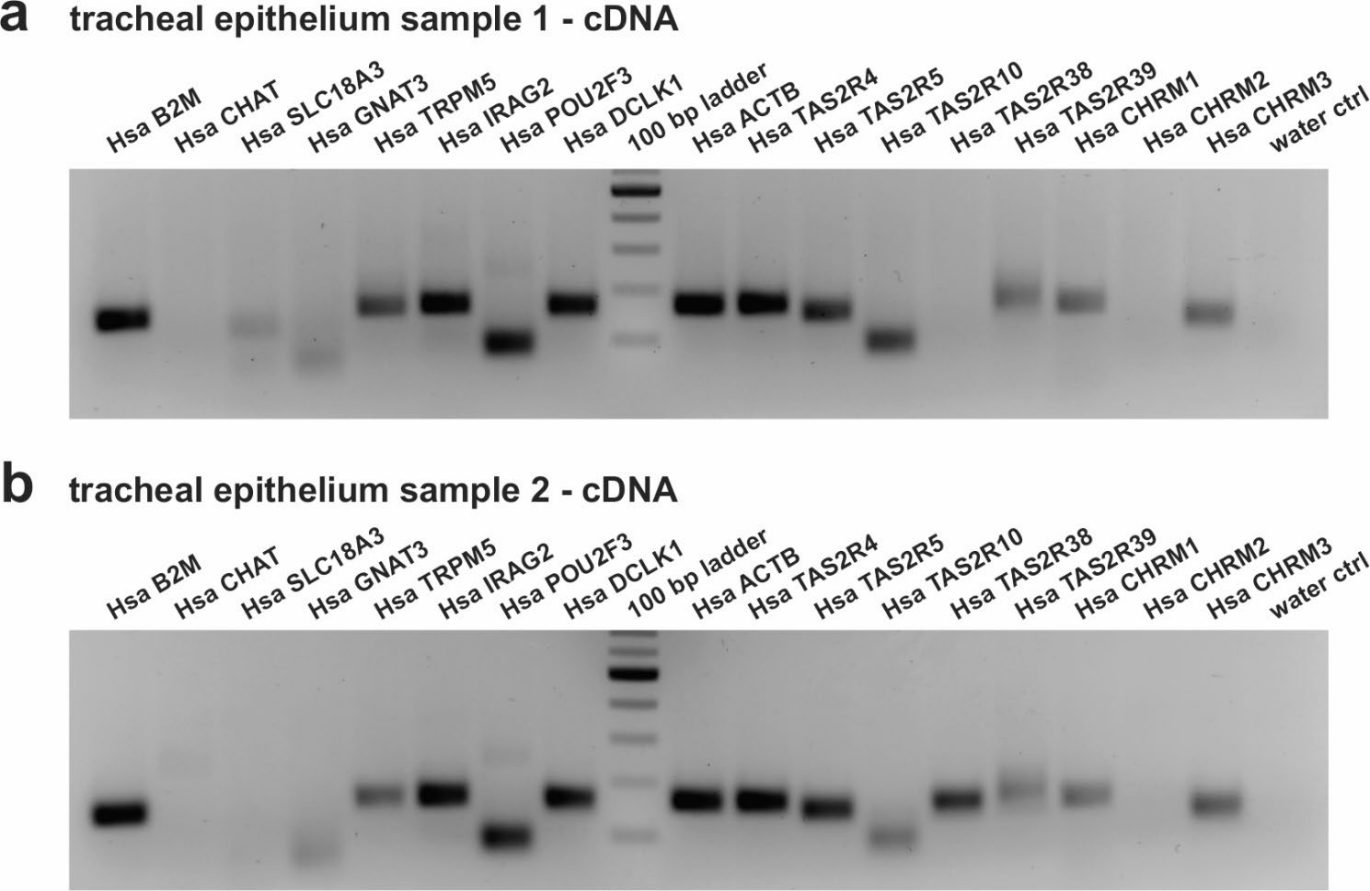
Gene transcription profile of human tracheal epithelium. Beta-microglobulin (B2M), actin beta (ACTB), both serving as controls, the tuft cell marker transcripts TRPM5, IRAG2, POU2F3, DCLK1 yielded a positive signal at the expected size. Further, the presence of taste receptor transcripts of TAS2R4, TAS2R5, muscarinic ACh receptors 1 and 3 (CHRM1/3) were verified. CHAT, SLC18A3, GNAT3, and CHRM2 yielded none or unspecific signals. TAS2R38 appeared in only one human sample, and TAS2R10/39 were also present in control reactions. Input controls of RNA without reverse transcription and genomic controls for each assay can be found in Suppl Fig. 2

Next, we extended our investigations on human RNA samples collected and purified from tracheal epithelium (Fig. 4a, b). Besides the range of tuft cell markers from our immunofluorescence analyses (CHAT, POU2F3, DCLK1; Fig. 2), we further detected IRAG2 (inositol 1,4,5-triphosphate associated protein 2), which has recently been demonstrated to be a gene expressed specifically in tuft cells [1, 10, 11]. Additionally, we were able to amplify TRPM5, and taste 2 receptor members 4 and 5. Transcription of TAS2R38 was only evident in one of the epithelial preparations, whereas the presence of TASR10 and TAS2R39 remains debatable given unexpected amplicons in our control (Suppl. Figure 2). Muscarinic AChR1 and 3 but not 2 were also detected. Surprisingly, CHAT, SLC18A3 (i.e., ‘VACHT’, vesicular acetylcholine transporter) and GNAT3 were either absent or remained below detection limits.

**Fig. 5:**
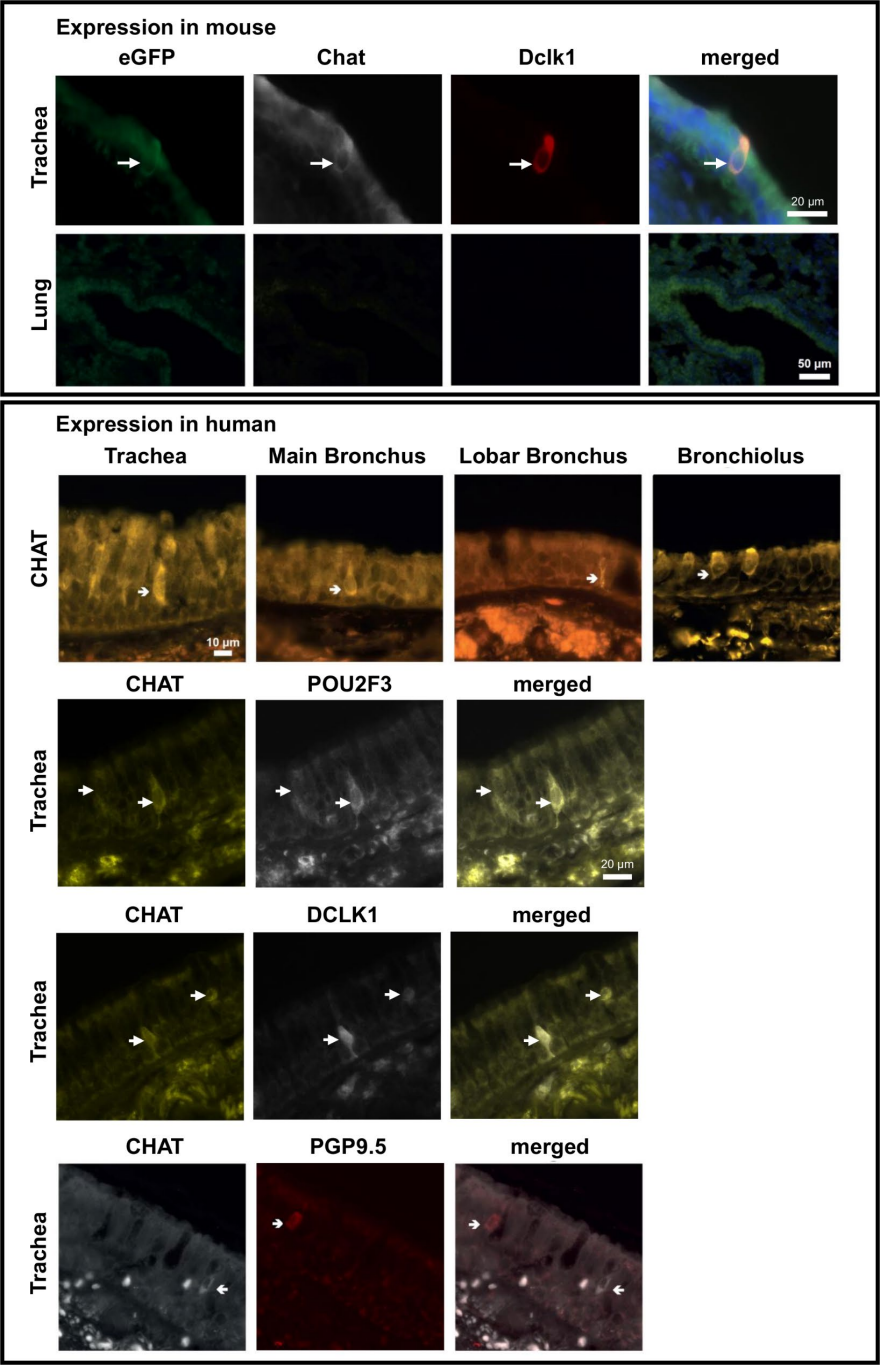
Immunofluorescence for CHAT in mouse trachea and human airways. Chat staining was observed in mouse trachea in eGFP^+^ Dclk1^+^ tuft cells but absent in mouse lung. In human samples CHAT was detected in the epithelium of the trachea, the main and lobar bronchus, as well as in bronchioles. CHAT staining was observed in POU2F3^+^ or DCLK1^+^ tuft cells. CHAT staining did not overlap with PGP9.5 staining of neuroendocrine cells

**Fig. 6:**
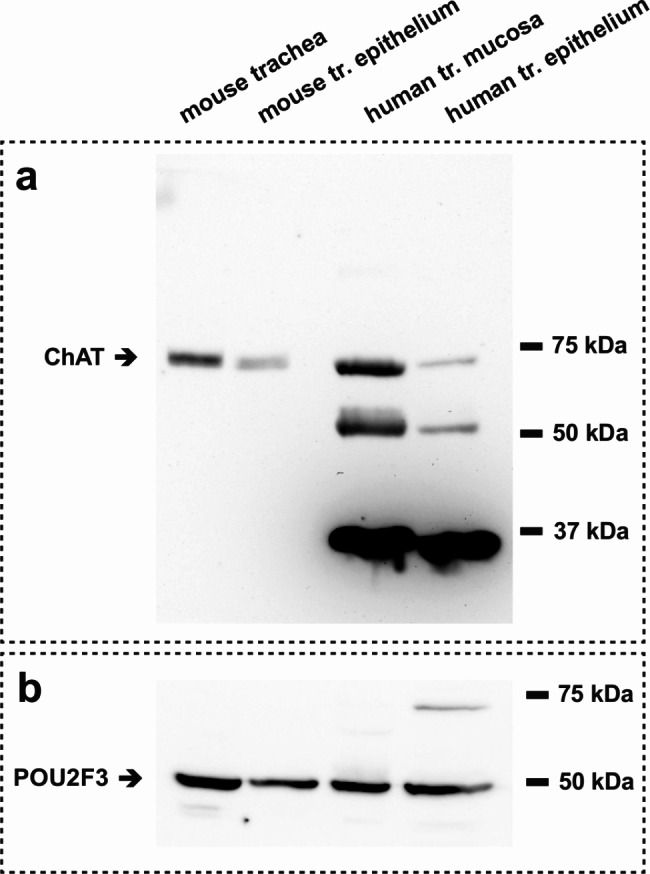
Immunoblot analysis of CHAT expression in mouse and human tissue samples. (**a**) Potential expression of choline acetyltransferase (CHAT) protein in samples from mouse tracheal homogenate, mouse tracheal epithelium, human tracheal mucosa and human tracheal epithelium. In all samples CHAT protein was present at the estimated molecular weight (pointing arrow). (**b**) The tuft cell marker POU2F3 (estimated size of approx. 50 kDa) was detected by immunoblot in all four samples

Given the importance of CHAT in the context of tuft cell signalling we attempted determining the presence of choline acetyltransferase protein by immunohistochemistry and immunoblotting (Figs. 5 and 6). Immunohistochemistry of mouse tracheal epithelium confirmed expression of Chat in tuft cells and showed specificity of the used antibody, since Chat staining was overlapping with GFP-immunofluorescence in brush cells of ChAT-eGFP mice [19]. Furthermore, the Chat^+^ cells were also positive for the tuft cell marker Dclk1 in mice (Fig. 5). In human airway sections we were able to detect CHAT in cells displaying the morphology of tuft cells in the tracheal epithelium as well as in the epithelium of the main and lobar bronchi, and in bronchioles. Co-localization of CHAT with POU2F3 or DCLK1 in the same cells confirmed that CHAT expressing cells were indeed tuft cells (Fig. 5). Moreover, CHAT^+^ cells were a population distinct from protein gene product 9.5 positive (PGP9.5^+^) cells, indicating that these CHAT^+^ cells are not overlapping with neuroendocrine cells.

Presence of choline acetyltransferase could be visualized in mouse total trachea and tracheal epithelium samples as well as in human tracheal mucosal and epithelium samples using immunoblotting (Fig. 6). In all four sample preparations a positive band for CHAT emerges in an estimated molecular weight range of 70 to 75 kDa. Additionally, we were able to detect the tuft cell marker POU2F3 in the same samples at an estimated molecular weight of approximately 50 kDa.

### Influence of tuft cells on mucociliary clearance in mouse and human tracheal epithelium

**Fig. 7:**
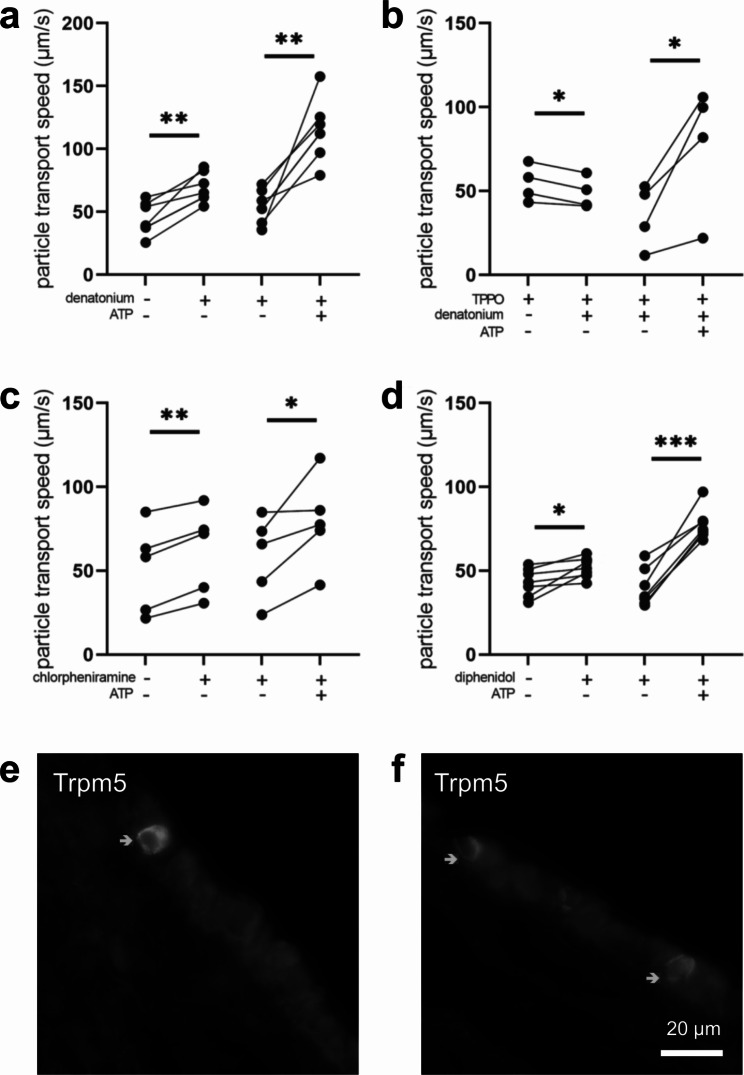
Bitter substances stimulate the particle transport speed (PTS) in the mouse trachea. (**a**) The bitter substance denatonium (1 mM) increased PTS (n = 7 tracheal pieces of 7 mice). (**b**) In the presence of the Trpm5 channel inhibitor TPPO (100 µM), denatonium decreased PTS (n = 4 tracheal pieces of 4 mice). (**c**) The bitter substance diphenidol (200 µM) increased PTS (n = 7 tracheal pieces of 6 mice). (**d**) The bitter substance chlorpheniramine (300 µM) increased PTS (n = 4 tracheal pieces of 4 mice). (a-d) ATP (100 µM), used as viability control, led to an increase in PTS. *: p < 0.05, **: p < 0.01, ***: p < 0.001. (**e-f**) Immunostaining for Trpm5 in mouse tracheal epithelium. Scale bar: 20 μm

In mice it has previously been shown that activation of tracheal tuft cells with the bitter substance denatonium, with *P. aeruginosa* quorum-signalling molecules or with bacterial formyl peptides stimulates the mucociliary clearance [11, 14, 21]. Here, we assessed the role of additional bitter substances on mucociliary clearance in mouse tracheal epithelium. First, we could confirm our previous finding that the bitter substance denatonium (1 mM) increased PTS significantly by 24.6 μm/s (Fig. 7a). This effect was tuft cell specific, since denatonium was decreasing PTS in the presence of the Trpm5 channel inhibitor TPPO by 5.8 μm/s (Fig. 7b). This shows that the observed denatonium-induced effect was indeed due to an activation of tuft cells, since expression of Trpm5 channels is restricted to chemosensory cells in the airway epithelium of mice [1, 7, 9, 11]. The bitter substances chlorpheniramine (300 µM) and diphenidol (200 µM), which both act on several Tas2R, such as Tas2r108, a tuft cell marker, Tas2r140 and Tas2r144 (chlorpheniramine), or Tas2r105, Tas2r108, Tas2r110, Tas2r122, Tas2r137 and Tas2r144 (diphenidol) [22], increased PTS to a similar extend, by 11.1 μm/s and by 10.9 μm/s, respectively (Fig. 7c, d), underlining the role for bitter taste receptors in acceleration of mucociliary clearance. ATP led to a significant increase in PTS in all experiments (Fig. 7), indicating that the tissue was still viable at the end of each experiment. In addition to the functional role of Trpm5 in denatonium-induced increase of PTS, we detected Trpm5 protein expression in mouse tracheal epithelium by immunohistochemistry (Fig. 7e, f), thereby strengthening our previous findings [23].

**Fig. 8:**
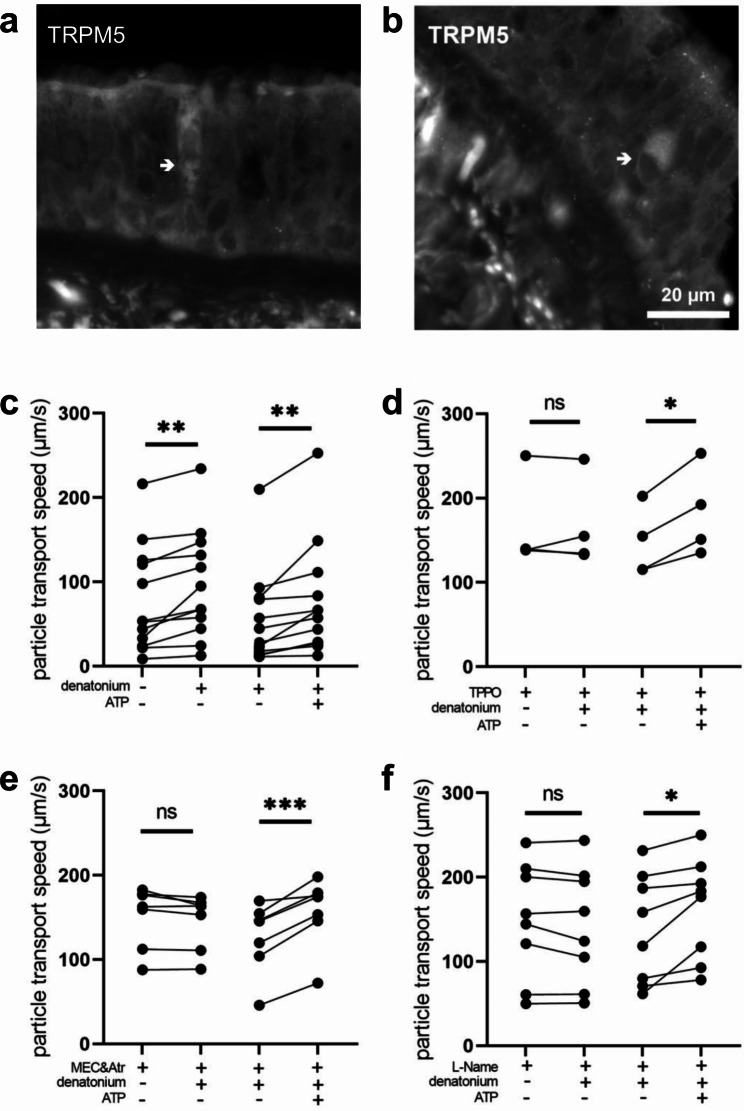
Denatonium induces tuft cell-mediated stimulation of particle transport speed (PTS) in human tracheal epithelium. (**a**) The bitter substance denatonium (1 mM) transiently increased PTS (n = 12 tracheal pieces of 7 body donors). (**b**) In the presence of the Trpm5 channel inhibitor TPPO (100 µM), denatonium did not alter PTS (n = 4 tracheal pieces of 3 body donors). (**c**) The cholinergic receptor antagonists mecamylamine (100 µM, nicotinic (n) AChR inhibitor) and atropine (50 µM, mAChR inhibitor) abolished the denatonium-induced PTS increase (n = 7 tracheal pieces of 5 body donors). (**d**) In the presence of the nitric oxide synthase inhibitor L-Name (20 µM), denatonium had no effect on PTS (n = 8 tracheal pieces of 6 body donors). (**a-d**) ATP (100 µM), used as viability control, led to a significant increase in PTS. *: p < 0.05, **: p < 0.01, ***: p < 0.001, ns: non-significant. (**e-f**) Immunofluorescence staining of TRPM5 in human tracheal epithelium. Scale bar: 20 μm

Since Trpm5 mediates tuft cell-dependent regulation of mucociliary clearance in mice, we investigated whether TRPM5 occurred in human tracheal epithelium on the protein level. The specificity of the TRPM5 antibody was validated on human taste bud samples (Suppl. Figure 3). Indeed, in addition to detecting TRPM5 transcripts in human tracheal epithelium, we were also able to detect TRPM5 by immunohistochemistry in tracheal sections (Fig. 8a, b), suggesting that TRPM5 might play a role in human tuft cell function. Next, we sought to investigate the influence of tracheal tuft cells on mucociliary clearance in native human tracheal epithelium. Upon addition of denatonium (1 mM), PTS increased by 17.4 μm/s (Fig. 8c). This increase in PTS was abolished in the presence of the TRPM5 channel inhibitor TPPO (100 µM, Fig. 8d).

Of note, the signalling molecule ACh, which has been shown to be released from tracheal tuft cells in mice [11, 14] seems to be involved in the denatonium-induced acceleration of PTS in humans, since the inhibitors of ACh receptors mecamylamine (nAChRs, 100 µM) and atropine (mAChRs, 50 µM) abolished the denatonium-induced effect (Fig. 8e). This indicates that human tuft cells are cholinergic and release ACh supporting a functional role for the CHAT protein detected by immunohistochemistry and immunoblotting (Figs. 5 and 6). In human upper airways, mucociliary clearance can be regulated by nitric oxide (NO) [24, 25], therefore we further investigated the role of NO in tuft cell mediated effects on mucociliary clearance. In the presence of the broad inhibitor of NO synthases (NOS) L-Name (20 µM), the denatonium-induced increase in PTS was abolished (Fig. 8f) indicating that stimulation of mucociliary clearance via the bitter signalling cascade was dependent on NO.

**Fig. 9:**
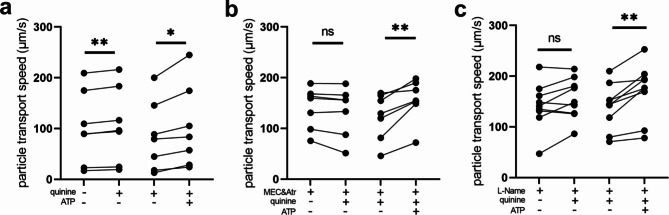
Quinine stimulates the particle transport speed (PTS) in human tracheal epithelium. (**a**) The bitter substance quinine (100 µM) transiently increased PTS (n = 7 tracheal pieces of 5 body donors). (**b**) In the presence of the cholinergic receptor antagonists mecamylamine (100 µM) and atropine (50 µM), quinine did not alter PTS (n = 7 tracheal pieces of 5 body donors). (**c**) In the presence of the nitric oxide synthase inhibitor L-Name (20 µM), quinine had no effect on PTS (n = 9 tracheal pieces of 6 body donors). (**a-c**) The viability control ATP (100 µM) led to a significant increase in PTS. *: p < 0.05, **: p < 0.01, ns: non-significant

Besides denatonium, which has been shown to act on several human bitter taste receptors, we used quinine as an alternative bitter taste receptor agonist. This agonist acts among others on the human TAS2R4 [26], the orthologue for the mouse Tas2r108, which is predominantly expressed in tuft cells [9, 11]. Quinine (100 µM) increased PTS in human tracheal epithelial preparations by 5.6 μm/s (Fig. 9a). This increase was also dependent on NO and ACh since it was abolished in the presence of mecamylamine and atropine (Fig. 9b) as well in the presence of L-Name (Fig. 9c). Application of ATP (100 µM) confirmed the viability of the tissue and demonstrated a robust increase in PTS by 26.6 μm/s in all series of experiments conducted on human tracheal preparations (Figs. 8 and 9). Taken together these results describe a scenario in which tuft cells play a role in the stimulation of mucociliary clearance in human tracheal epithelium involving NO and cholinergic signalling.

## Discussion

Tuft cells in the airways have emerged as crucial regulators of innate immune processes in mice [5]. While tuft cells have also been described in recent RNAseq studies of human airway epithelium [2–4], it remained yet unclear whether human tuft cells also display chemosensory and cholinergic traits (as their mouse homologs) and whether they have similar functions in innate immunity. We here detected tuft cells throughout the airways and delineated a cholinergic function for tuft cells in the regulation of mucociliary clearance, a fundamental innate immune process of the airways.

In human airways, tuft cells have long been described morphologically in the trachea and bronchi [27, 28]. Under pathophysiological conditions they have further been detected in the alveoli of children [29, 30]. We identified tuft cells by the expression of their marker protein POU2F3 [4] throughout the conductive lower airways from the trachea to the bronchioles. They appeared to be absent in the human alveolar regions under physiological conditions. Transcripts of IRAG2, POU2F3, DCLK1, and TRPM5 were present in human tracheal epithelium, and protein expression of POU2F3, DCLK1 and CHAT, established markers for mouse tuft cells, has been confirmed by immunofluorescence analysis in various segments of the lower respiratory tract. These data delineate the presence of tuft cells with cholinergic features in the human lung.

Evidence suggesting the presence of chemosensory cells in human airways solely by means of RNAseq needs to be considered with precaution. In one study conducted with human bronchial primary epithelial cultures tuft cells clustered with neuroendocrine cells [2], while in another study using human tracheal samples tuft cells, neuroendocrine cells and ionocytes emerged as distinct cell populations. In the latter study, tuft cells expressed POU2F3 [4] and therefore we consequently used POU2F3 for the identification of tuft cells in our study. Moreover, we found that CHAT expressing cells represented POU2F3 + and DCLK1 + cells given the overlap in the immunolabelling for these proteins. Strikingly, these cells were not identical to neuroendocrine cells, since CHAT and PGP9.5 immunostaining did not overlap. This further strengthens the assumption that tuft cells and neuroendocrine cells represent two distinct cell populations with most probably different functions. Since Chat is a marker for mouse tracheal epithelial cells [7, 9, 11], and POU2F3 is a marker for human tuft cells [7, 9, 11] it is likely that the CHAT^+^ cells co-expressing POU2F3 in the human tracheal samples are also tuft cells. In addition to the paracrine effects of ACh on ciliated cells, the presence of mAChR1 and 3 transcripts in human tracheal epithelium as well as in mouse tracheal tuft cells opens the possibility for a potential autocrine signalling as this has been previously described for mouse tuft cells [11, 14]. Since cholinergic neurons are located in the tracheal adventitia, and this was not included in our samples, we can exclude that intraepithelial nerves contribute to the mAChR transcripts detected in our study. In mice, ACh is released from tuft cells in a Trpm5-dependent manner and elicits important paracrine effects such as an increase in mucociliary clearance or an induction of neurogenic inflammation [11, 12, 14]. In support of this observation, we could here demonstrate that tuft cell stimulation by bitter substances in the human trachea plays a role in stimulating mucociliary clearance and involves cholinergic signalling.

In human nasal airway epithelium, TAS2R are present in chemosensory cells and possibly also in ciliated cells [18, 31]. In the human tracheal epithelium we were able to detect TAS2R4 transcripts, the orthologue for the mouse tuft cell marker Tas2r108, whereas transcription of TAS2R38, the receptor detected in human nasal ciliated cells [18], was less explicit. Detection of all potential bitter taste receptor transcripts in our study might be limited by the quality of RNA extracted from the body donors. Previous studies found bitter taste receptors also in ciliated epithelial cells obtained from tracheal and bronchial samples and ciliary beat frequency was increased upon stimulation with the bitter substance denatonium [17]. Denatonium has been shown to act on several human bitter taste receptors, among them the TAS2R4, an orthologue to the mouse Tas2r108, the hallmark receptor for mouse tracheal tuft cells [22, 26]. Interestingly, the observed stimulation of mucociliary clearance after application of denatonium to human tracheal epithelium from body donors, was dependent on TRPM5, which we detected on protein level. Expression of Trpm5 has been attributed exclusively to chemosensory cells in mouse airway epithelia [1, 7, 9–11, 32]. The scarcity of TRPM5 staining in our human tracheal samples suggests that TRPM5 expression is limited to a rare epithelial cell type such as tuft cells. In support, TRPM5 was listed as a marker for human airway tuft cells (Suppl. Material in Deprez et al. [4]). Our data provide evidence for functional TRPM5 in human tracheal epithelium and point towards its role in activation of mucociliary clearance after stimulation of the bitter taste signalling cascade as this has recently been demonstrated in mice [11]. The underlying mechanism involves NO in addition to ACh, as the denatonium-induced increase in mucociliary clearance was reduced upon inhibition of NOS or antagonising AChRs.

In mouse trachea, the activation of nAChRs and mAChRs is well established as a stimulator of mucociliary clearance, measured as an increase in PTS [33, 34]. Moreover, we have shown previously, that ACh released from a non-neuronal source in the mouse trachea modulates mucociliary clearance by activating transepithelial ion transport processes via mAChRs and nAChRs [35–38]. Recently, mouse tracheal brush cells have been identified as the source for the non-neuronal cholinergic regulation of transepithelial ion transport [15]. Here, we observed that the regulation of the mucociliary clearance in the human trachea was also dependent on cholinergic signalling, thus it is likely that this effect was mediated by cholinergic tuft cells that are equipped with the bitter transduction cascade. These findings, together with the presence of CHAT in POU2F3 + cells, suggests that human tuft cells are able to synthesize ACh, which then acts in a paracrine manner to stimulate mucociliary clearance. In human upper airways ACh is a known regulator of mucociliary clearance [24, 39, 40], yet the source of ACh-release was not addressed in the studies. Besides ACh, a production of NO in the epithelium leads to an increase in mucociliary clearance [18, 24, 25]. Since this was attributed to ciliated cells [18], it is tempting to speculate that tuft cell-released ACh acts on ciliated cells in a paracrine manner, thereby stimulating NOS in these cells. Supportive of this hypothesis is a recent finding in mouse tracheas showing that activation of tuft cells leads to release of ACh, which excites neighbouring cells and initiates a Ca^2+^ wave through gap junction signalling, reaching also distant ciliated and secretory cells [15]. Since NOS can be activated by binding of Ca^2+^ to calmodulin [41], tuft cell-released ACh might lead to NO production by an increase of intracellular Ca^2+^ in ciliated cells. Taken together, our data support, a tuft cell-dependent stimulation of mucociliary clearance that was mediated by NO and ACh signalling. This is suggestive for a role of human tracheal epithelial cells in the induction of innate immune processes, since an increase in mucociliary clearance is a crucial step in preventing infections by transporting bacteria out of the airways.

## Conclusion

In summary, our study provides evidence for cholinergic traits in human tuft cells and delineates the distribution of these cells in the lower respiratory tract: in the trachea, in the main and lobar bronchi, as well as in the bronchioles. We clearly show that these cholinergic aspects of human tuft cells are functional and that also TRPM5 is present in human tracheal epithelium. Cholinergic and TRPM5 signalling was involved in stimulating mucociliary clearance, a fundamental innate immune process of the airways needed to transport inhaled pathogens out of the airways, thereby preventing infections. Therefore, it is tempting to speculate that human tuft cells in the lower airways might play a role in eliciting protective innate immune processes, such as recruitment and activation of immune cells similar to that observed after activation of mouse tracheal tuft cells by bacteria [12], that might be crucial to combat bacterial infections as has been shown for the lower airways in mice [12].

### Electronic supplementary material

Below is the link to the electronic supplementary material.

Supplementary Material 1

## Data Availability

The datasets included in this publication are available from the corresponding author on reasonable request.

## Abbreviations

ACh: Acetylcholine
Actb/ACTB: Mouse/human actin beta
B2m/B2M: Mouse/human beta microglobulin 2
CBF: Ciliary beat frequency
ChAT/CHAT: Mouse/human choline acetyltransferase
Chrm1/CHRM1: Mouse/human cholinergic receptor muscarinic 1
IRAG2: Human inositol 1,4,5-triphosphate receptor associated 2
Gnat3/GNAT3: Mouse/human G protein subunit alpha transducing
HE: Hematoxylin/eosin
mAChR: Muscarinic acetylcholine receptor
nAChR: Nicotinic acetylcholine receptor
NO: Nitric oxide
NOS: Nitric oxide synthase
PGP9.5: Protein gene product 9.5
Pou2f3/POU2F3: Mouse/human POU class 2 homeobox 3
PTS: Particle transport speed
Slc18a3/SLC18A3: Mouse/human solute carrier family 18 member A3
T2r105: Mouse taste 2 receptor member 105
TAS2R4: Human taste 2 receptor member 4
Trmp5/TRPM5: Mouse/human transient receptor potential cation channel subfamily M member 5
TPPO: Triphenylphosphine oxide
