# Supplementary material for: Human airway tuft cells influence the mucociliary clearance through cholinergic signalling
Source: Respir Res. 2023 Nov 4;24:267. doi: 10.1186/s12931-023-02570-8 (PMC10625704; doi:10.1186/s12931-023-02570-8)
Supplement: Supplementary file 1 — Supplementary Material 1 [file 12931_2023_2570_MOESM1_ESM.pdf]

Suppl. Table 1: Primer sequences

gene name	species	forward	reverse	size	nucelotide reference	
B2m	mouse	ATTCACCCCCACTGAGACTG	GCTATTTCTTTCTGCGTGCAT	192 bp	NM_009735.3	
Chat	mouse	GACCAGCTAAGGTTTGCAGC	CAGGAAGCCGGTATGATGAGA	163 bp	NM_009891.2	
Slc18a3	mouse	TTGATCGCATGAGCTACGAC	AGGCTCCTCGGGATACTTGT	188 bp	NM_021712.3	
Tas2r105	mouse	GACTGGCTTCCTTCTCATCG	GCAAACACCCCAAGAAAA	284 bp	NM_020501.1	reverse oligo mut
Tas2r108	mouse	TGGATGCAAACAGTCTCTGG	GGTGAGGGCTGAAATCAGAA	158 bp	NM_020502.1	
Trpm5	mouse	TGAGGAACGACCTTTGGCTA	ACACGGATCTTGGTGGATGT	183 bp	NM_020277.2	
Gnat3	mouse	TCATCCATAAGAATGGTTACAGC	CCCACAGTCGTTTAATGATTTC	231 bp	NM_001081143.1	
Chrm1	mouse	AGAAGAGGCTGCCACAGGTA	CAGACCCCACCTGGACTTTA	198 bp	NM_001112697.1	
Chrm2	mouse	GAATGGGGATGAAAAGCAGA	GCAGGGTGCACAGAAGGTAT	192 bp	NM_001411690.1	forward oligo mut
Chrm3	mouse	CACAGCCAAGACCTCTGACA	ATGATGTTGTAGGGGGTCCA	222 bp	NM_033269.4	
Actb	mouse	GTGGGAATGGGTCAGAAGG	GGCATACAGGGACAGCACA	300 bp	NM_007393.5	
B2M	human	GGCATTCCTGAAGCTGACAG	TGGATGACGTGAGTAAACCTG	135 bp	NM_004048.4	
CHAT	human	AAACCTACCTGATGAGCAACC	GTTGTAGCAGGCACCATACC	116 bp	NM_020549.5	
SLC18A3	human	CTCATGCTAGACCCCTACATTG	TCGTATGCTTCATCCACGTG	106 bp	NM_003055.3	
GNAT3	human	TGAAAGCCATGACTACCCTTG	AGCCAGTTGAGGTGTCATG	119 bp	NM_001102386.3	
TRPM5	human	AGAACTTCCTGAGCAAGATGG	TGACTCCAGACACTTGATGC	167 bp	XM_047426859.1	
POU2F3	human	AGGAGAAGCGAATCAACTGC	GGGAACAGGATGACGTTACTG	150 bp	NM_001244682.2	
IRAG2	human	AGCAAGAATACATCAGACACCC	CCAGAGATAAGGCATCCAGC	77 bp	NM_001366544.2	
DCLK1	human	CATTGCAGAGACTGGATACGG	ACCTGCCCCATCAAAATCTG	144 bp	NM_001330071.2	
ACTB	human	ACCTTCTACAATGAGCTGCG	CCTGGATAGCAACGTACATGG	148 bp	NM_001101.5	
TAS2R4	human	GTGGTTTCTTTGGTCTTGAGC	CCTACATGAGCTTCCGTCTG	149 bp	NM_016944.2	
TAS2R5	human	CTCTGGGATGCTGATTGTCTC	CAGGTGAAGTAAGAGGAAGCAG	136 bp	NM_018980.3	
TAS2R10	human	CATTTCCCTTTGGAGACACAAC	ATGAGCTTCTGTGTTGGAGTC	76 bp	NM_023921.2	
TAS2R38	human	GTTGACTCTAACTCGCATCCG	GCCTCTTCACTACATCCCAAA	140 bp	NM_176817.5	
TAS2R39	human	ATTACTGGATTGATACCCTGGC	TGTTTTCTTAGTGGAGTTGGAGG	138 bp	NM_176881.2	
CHRM1	human	ATGCCTCCGTCATGAATCTG	CAGAGCACAAAGGAAACCAG	142 bp	NM_000738.3	
CHRM2	human	CACAAAACCTCTGACCTACCC	ACAGTTCTCACCCCTACAATG	138 bp	NM_001006630.2	
CHRM3	human	CAGATGGTTAGGAGAAGTGAGC	AGGGTGACTAGGAGAATAGGG	118 bp	NM_001375978.1	

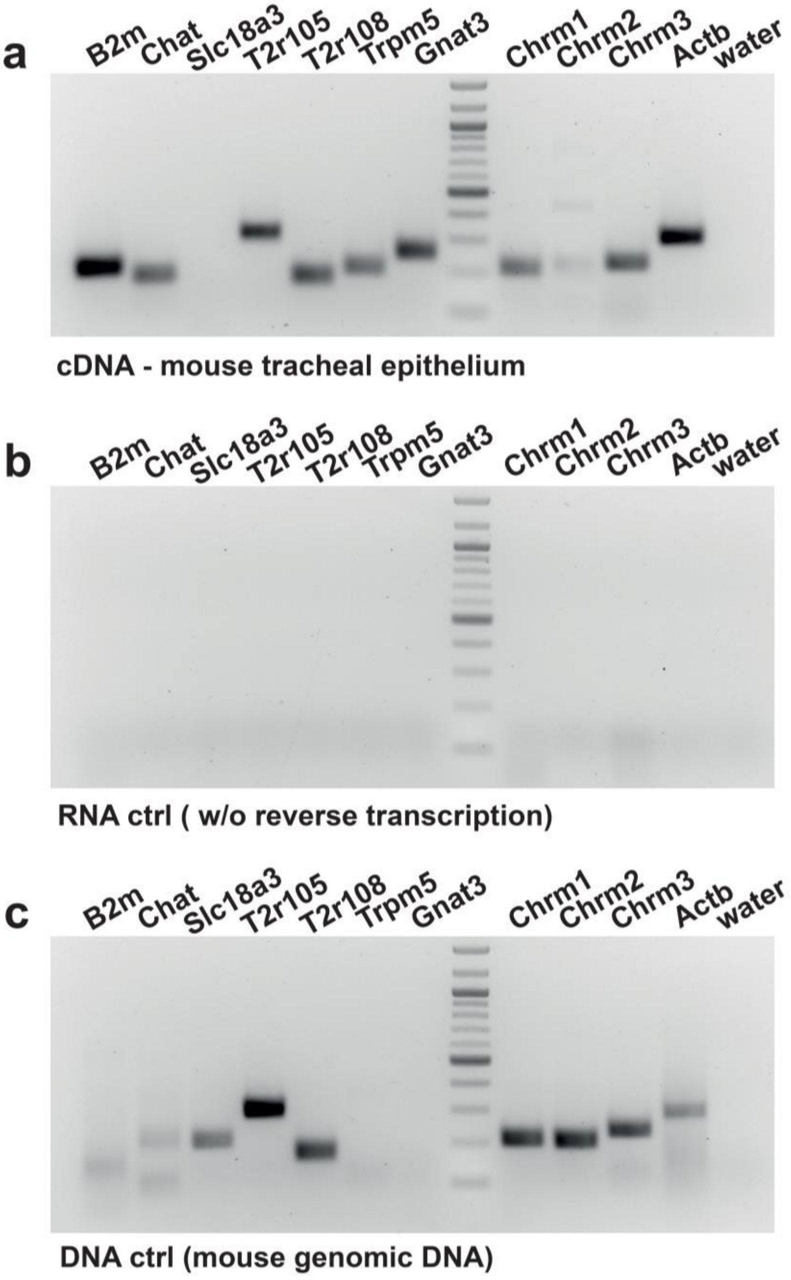

Suppl. Fig. 1: Qualitative analysis of the gene transcription profile of mouse tracheal epithelium including assay controls. All pictures represent endpoint PCR results separated on an agarose gel. (a) cDNA of a DNaseI-treated RNA sample of mouse tracheal epithelium indicates detectable transcription of all tested genes despite solute carrier family 18 member a3 transcripts. (b) Specificity of amplified gene transcripts was assayed applying amplification strategies to DNaseI-treated but not reversely transcribed RNA to estimate potential contamination by genomic DNA during the process of RNA extraction. The absence of any amplicon suggests that results in (a) indicate the successful reverse transcription of RNA from tracheal epithelium. (c) Qualitative assessment of potential interference by genomic DNA during the amplification process leads to potentially unspecific bands in cases in which gene assays could have not been set up in an intron spanning fashion, this included Slc18a3, both taste receptor genes and all three muscarinic ACh receptor genes. The coding sequence of these genes is present on only one exon.

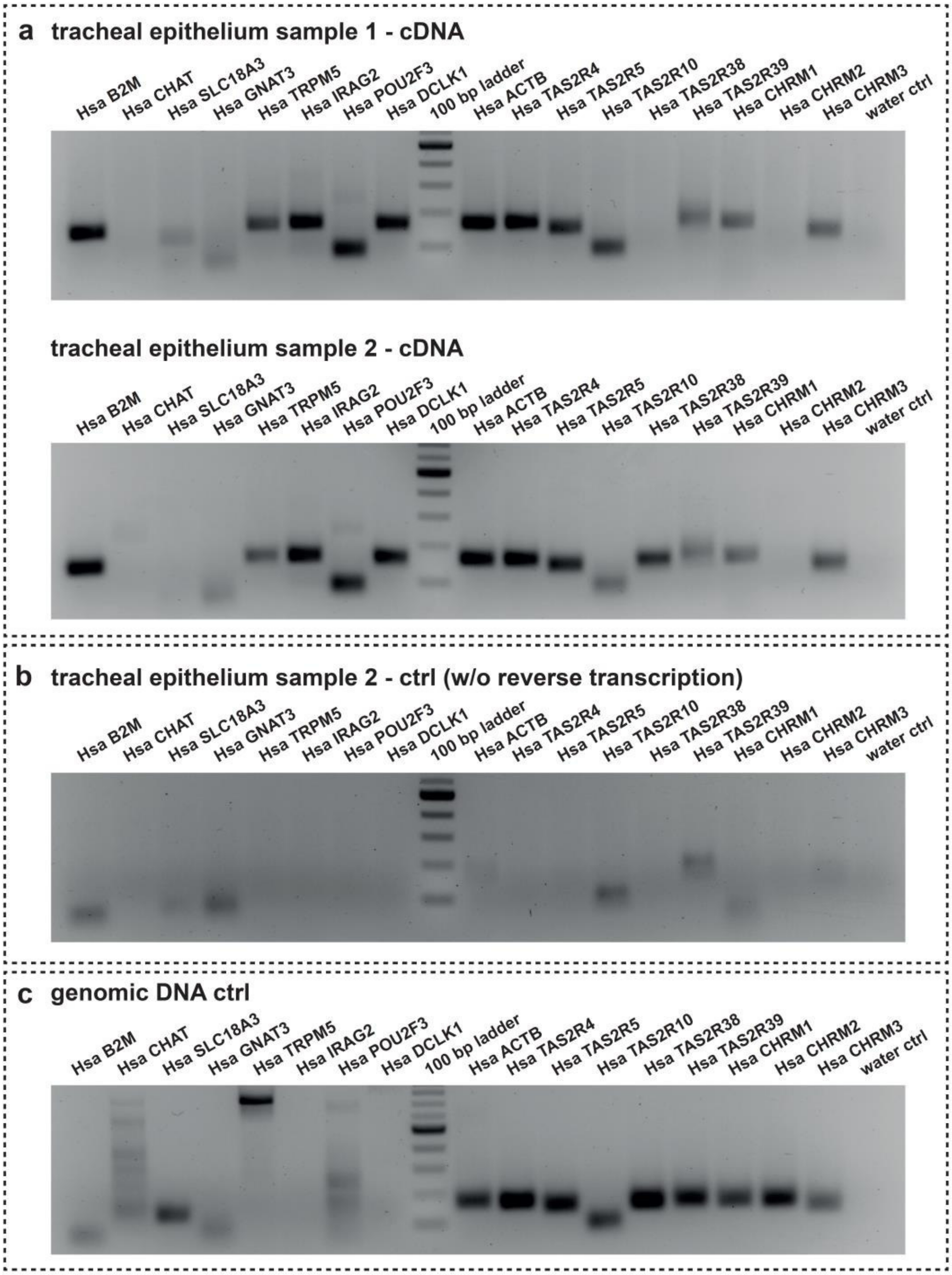

Suppl. Fig. 2: Qualitative analysis of the gene transcription profile of human tracheal epithelium including assay controls. All pictures represent endpoint PCR results separated on an agarose gel. (a) Set of tested genes using DNaseI-treated RNA samples isolated from two human body donors that have been transcribed into cDNA (Fig. 4). (b) Control reaction applying DNaseI-treated RNA samples that have not been reversely transcribed into cDNA to assess unspecific amplification, i.e., false positive reactions due to amplification from, for example, genomic DNA contamination. (c) Application of amplification strategies using human genomic DNA to compare the presence or absence of amplicons from transcripts deriving from genes in which a selection of intron-spanning assays was not possible, e.g., SLC18A3, all depicted taste receptors as well as muscarinic ACh receptors. Comparison of (a), (b) and (c) reveals that testing for TAS2R10 and TAS2R39 resulted in an unspecific signal, yet different from genomic contamination due to size differences of its amplicons in (a).

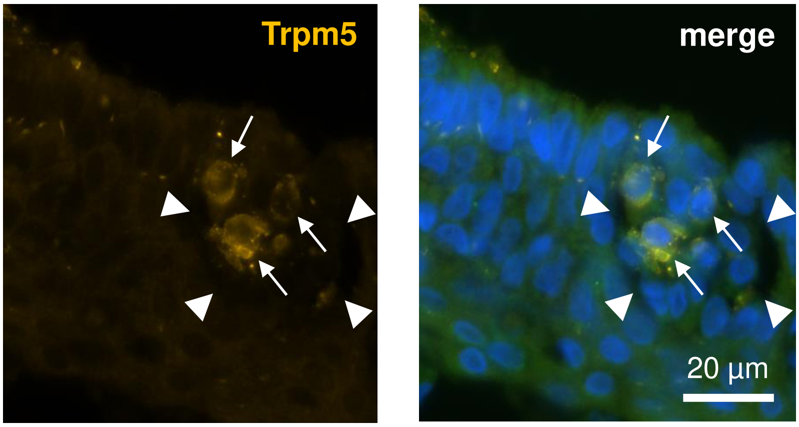

Suppl. Fig. 3: Taste bud in a human circumvallate papilla. TRPM5⁺ cells are visible in a taste bud. The arrowheads outline the taste bud. Immunofluorescence staining for TRPM5 (yellow, arrows) in epithelial cells of a taste bud. Merge: DAPI for visualisation of the nuclei, tissue autofluorescence in the green emission channel for representation of the epithelium. Scale bar: 20 µm
